# BDP1 as a biomarker in serous ovarian cancer

**DOI:** 10.1002/cam4.5388

**Published:** 2022-10-28

**Authors:** Stephanie Cabarcas‐Petroski, Gabriella Olshefsky, Laura Schramm

**Affiliations:** ^1^ Biology Department, Pennsylvania State Beaver Pennsylvania State University Monaca Pennsylvania USA; ^2^ Garden City High School Garden City New York USA; ^3^ Biology Department St. John's University Queens New York USA

**Keywords:** BDP1, ovarian cancer biomarkers, RNA polymerase III, serous ovarian cancer, TFIIIB

## Abstract

**Background:**

TFIIIB, an RNA polymerase III specific transcription factor has been found to be deregulated in human cancers with much of the research focused on the TBP, BRF1, and BRF2 subunits. To date, the TFIIIB specific subunit BDP1 has not been investigated in ovarian cancer but has previously been shown to be deregulated in neuroblastoma, breast cancer, and Non‐Hodgkins lymphoma.

**Results:**

Using *in silico* analysis of clinically derived platforms, we report a decreased BDP1 expression as a result of deletion in serous ovarian cancer and a correlation with higher and advanced ovarian stages. Further analysis in the context of TP53 mutations, a major contributor to ovarian tumorigenesis, suggests that high BDP1 expression is unfavorable for overall survival and high BDP1 expression occurs in stages 2, 3 and 4 serous ovarian cancer. Additionally, high BDP1 expression is disadvantageous and unfavorable for progression‐free survival. Lastly, BDP1 expression significantly decreased in patients treated with first‐line chemotherapy, platin and taxane, at twelve‐month relapse‐free survival.

**Conclusions:**

Taken together with a ROC analysis, the data suggest BDP1 could be of clinical relevance as a predictive biomarker in serous ovarian cancer. Lastly, this study further demonstrates that both the over‐ and under expression of BDP1 warrants further investigation and suggests BDP1 may exhibit dual function in the context of tumorigenesis.

## Graphical abstract

Using *in silico* analysis, the data suggests that BDP1, a subunit of the RNA polymerase III specific transcription factor TFIIIB, could be of clinical relevance as a predictive biomarker in serous ovarian cancer.

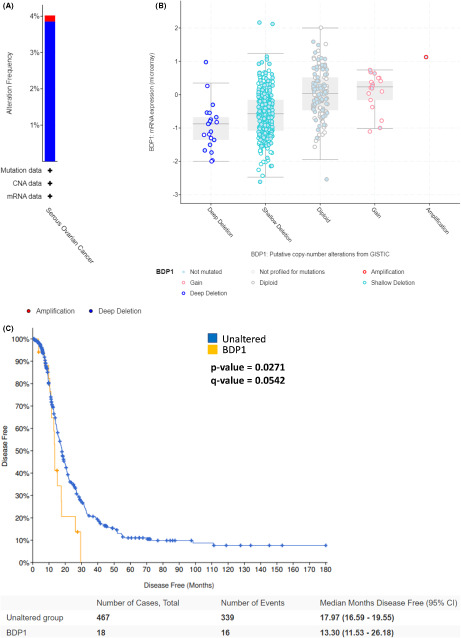

## 1 INTRODUCTION

In the United States, ovarian cancer ranks fifth in cancer‐related deaths^1^ with approximately 19,880 new diagnoses and 12,810 deaths anticipated for 2022.^1^ Although ovarian cancer is the most lethal gynecological cancer,^2^ screening remains largely ineffective as a preventative measure. Diagnosis frequently occurs at an advanced stage requiring a transvaginal ultrasound (TVUS) and the cancer antigen 125 (CA‐125) blood test.^2^ The CA‐125 blood test has been the most utilized clinical tool for screening, detecting, and managing ovarian cancer for over four decades with approximately 92% of advanced‐stage serous ovarian cancers exhibiting elevated levels.^3^ Nearly all ovarian tumors originate from epithelial cells, stromal cells, and germ cells^4^ with over 90% of malignant ovarian tumors having an epithelial origin. Stromal tumors make up 5%–6% and germ cell tumors comprise 2%–3% of ovarian tumors.^4^ The five principal histotypes are high‐grade serous (HGSOC), clear cell (CCOC), endometrioid (ENOC), mucinous (MOC), and low‐grade serous (LGSOC).^5^ Approximately 23% of all ovarian cancers have a hereditary component^6^ and both familial and sporadic ovarian cancers have been associated with mutations in BRCA1 or BRCA2.^7^ The lifetime risk of developing ovarian cancer is 40–45% for women with mutations in BRCA1 and 15–20% harboring BRCA2 mutations.^8^ Additional genetic alterations in cellular recombination and repair pathways have been identified in ovarian cancer, including TP53, PIK3CA, and PTEN.^6^, ^8^ Interestingly, BRCA1,^9^ TP53,^10^, ^11^ PTEN,^12^, ^13^ and the PI3K signal transduction pathway^13^ have been shown to specifically deregulate RNA polymerase III transcription in a variety of cancers.^14^, ^15^, ^16^

Eukaryotic RNA polymerases (pol), I ‐ III, regulate cellular growth,^17^ with RNA pol III regulating the transcription of untranslated small RNA molecules involved in processing and translation, thus, controlling a cell's biosynthetic capacity.^17^ Accurate transcription initiation by RNA pol III requires gene‐specific and general transcription factors including the RNA pol III specific TFIIIB complex.^17^ To date, two forms of TFIIIB have been characterized in humans and both require BDP1 and TBP.^18^, ^19^ The TFIIIB subunits, BRF1,^20^, ^21^, ^22^, ^23^ required for gene‐internal promoters, and BRF2,^12^, ^16^, ^24^, ^25^, ^26^, ^27^, ^28^, ^29^, ^30^, ^31^, ^32^, ^33^ required for gene‐external promoters, distinguish the two forms and have been well‐studied in various human cancers.

Recently, the BDP1 subunit of TFIIIB, has been identified as altered in human cancers.^34^, ^35^, ^36^ Specifically, in colorectal cancer, BDP1 somatic frameshift mutations were identified, *n =* 98, but clinical outcome data were not reported.^37^ In neuroblastoma, two BDP1 variants were identified to be associated with poor clinical outcomes^36^ and recently, BDP1 expression has been correlated with clinical outcomes in non‐Hodgkin lymphoma (NHL)^35^ and breast cancer.^34^ These recent BDP1 clinical cancer studies prompted our investigation of BDP1 alterations and expression in ovarian cancer.

Using open‐access clinically derived platforms, we analyzed BDP1 alterations in ovarian cancer samples *in silico*. The major advantage of using multiple bioinformatics platforms that utilize clinical samples to analyze BDP1 in ovarian cancer is that each platform employs various algorithms to determine statistical significance and confirm results using multiple analyses. Using this approach, we report that the BDP1 alterations identified in ovarian cancer were deep deletions with decreased expression correlating with increased serous ovarian cancer stage similar to known critical cancer drivers, BRCA1 and BRCA2. Interestingly, in the context of TP53 mutations, serous ovarian cancer patients with TP53 mutations displayed high BDP1 expression correlating with an unfavorable overall survival. These BDP1 alterations negatively impacted disease‐free progression in patients with ovarian cancer as well. Lastly, in patients treated with both platin and taxane, BDP1 expression was significantly decreased at 12‐month relapse‐free survival and a ROC analysis suggest a role for BDP1 as a predictive biomarker. This is the first study to implicate BDP1 in serous ovarian cancer and the first study to demonstrate varied expression for BDP1 in human cancer dependent on the mutation profile. These data suggest additional studies are warranted to evaluate the clinical use of BDP1 as a predictive biomarker in serous ovarian cancer, especially by stage and mutational profile.

## 2 MATERIALS AND METHODS

### 2.1 Identification of BDP1 alterations in ovarian cancer using the cBioPortal Platform

The cBioPortal is an open‐source multi‐cancer genomics and clinical dataset analysis.^38^, ^39^ Using the cBioPortal Platform, we queried for BDP1 alterations (June 2021 – April 2022) in the TCGA Firehouse Legacy Ovarian Serous Cystadenocarcinoma dataset, containing samples derived from 594 patients, Table 1.^40^ *P*‐values are derived from the Log Rank test and the *q*‐values are derived from the Benjamini‐Hochberg False Discovery Rate (FDR) correction procedure.

**TABLE 1:** A list of public datasets used in this study. Hyperlinks to datasets and study descriptions are provided

Dataset/Description	Reference
Ovarian Serous Cystadenocarcinoma (TCGA, Firehose Legacy; previously known as the TCGA provisional dataset)	^40^
GSE26193 Transcriptome analysis of high‐grade human ovarian adenocarcinomas	^41^
GSE63885 Gene expression profiling in ovarian cancer	^42^

### 2.2 Analysis of BDP1 expression in ovarian cancer using Gene Expression Profiling Interactive Analysis (GEPIA)

GEPIA is built by the HTML5 and JavaScript libraries, including jQuery and Bootstrap. For expression analyses, the GEPIA platform uses the TCGA and GTEx gene expression data re‐computed from raw RNA‐Seq data by the UCSC Xena project based on a consistent workflow, detailed in the help section of the GEPIA platform.^43^, ^44^ Both the TCGA and GTEx data used by GEPIA are derived from normal and tumor samples. For expression analyses, the log_2_FC cutoff used is 1.0, and the *p*‐value cutoff is 0.01. The matched normal analysis was performed using TCGA tumors versus TCGA normal and GTex normal. The log_2_ (TPM + 1) transformed expression data were used for plotting. For violin plots of cancer stage expression presented in Figure 3, analyses were performed using TCGA tumors versus TCGA normal and GTEx normal. The GEPIA platform utilizes pathological stages based on the TCGA clinical annotation.^44^, ^46^ The log_2_ (TPM + 1) transformed expression data was used for plotting, and a one‐way ANOVA analysis was performed. F and Pr(>F) values are denoted for each gene analyzed.^43^, ^44^ The GEPIA platform was accessed from November 2021–to April 2022.

### 2.3 BDP1 overall and progression‐free survival curves in ovarian cancer using Kaplan–Meier Plotter

We analyzed overall and progression‐free survival for high and low BDP1 (probe 226290_at) mRNA expression by stage and TP53 mutation status using the Kaplan–Meier Plotter (http://kmplot.com/analysis/) for ovarian cancer^48^; accessed June 2021 – April 2022. Parameters used in analyses included best cutoff, hazard ratio (HR) with 95% confidence intervals (CIs), log‐rank *p*‐value, biased arrays were excluded, and JetSet best probe was selected.^48^ GSE26193 and GSE63885, Table 1, were the datasets screened using Kaplan–Meier Plotter.

### 2.4 Analysis of BDP1 as a predictive biomarker using ROC Plot

ROC plotter (http://www.rocplot.org) is a receiver operating characteristic (ROC) tool for meta‐analysis‐based discovery and validation of survival biomarkers.^49^ The platform links gene expression and response to therapy using transcriptome data of 2369 ovarian cancer patients.^49^ Ovarian cancer dataset samples were divided into responder and nonresponder groups based on their clinical characteristics. Responders and nonresponders were compared using the Mann–Whitney test and the ROC test in the R statistical environment using Bioconductor libraries.^49^ The cutoff for p values was set at *p* < 0.05, and only results with a 5% false discovery rate (FDR) were considered significant.^49^ We queried the ROC plotter platform to predict BDP1 (probe 226290_at), TP53 (probe 201746_at) and NCOR2 (probe 207760_s_at) expression in serous ovarian cancer patients in response to chemotherapy; accessed January 2022 – August 2022.

## 3 RESULTS

### 3.1 Correlation between BDP1 and disease‐free progression and overall survival in ovarian cancer

**FIGURE 1:**
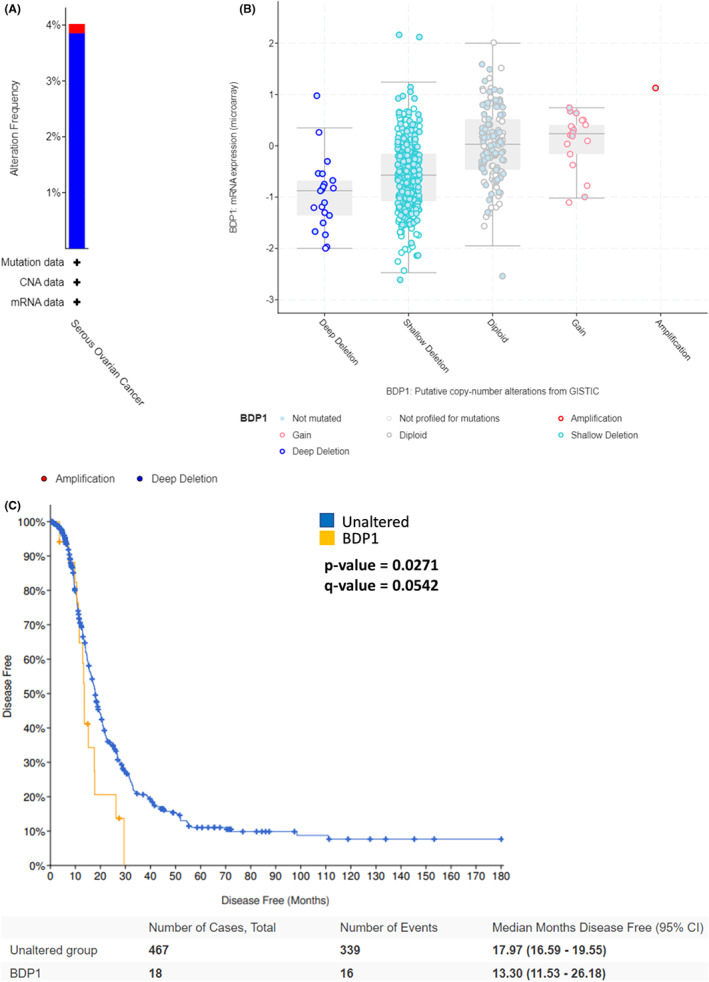
BDP1 alterations correlate with disease‐free progression and overall survival in ovarian cancer. (A) Using the cBioPortal platform,^38^, ^50^ we queried the TCGA Ovarian Serous Cystadenocarcinoma dataset (TCGA, Firehose Legacy), *n* = 594 patients and found twenty‐three cases of BDP1 homodeletions, 3.85% alteration frequency, and one instance of BDP1 amplification, 0.167% alteration frequency. (B) BDP1 mRNA expression from microarray versus BDP1 copy number detail the significance of BDP1 deep deletions. (C) BDP1 alterations impact disease‐free progression in patients with ovarian cancer. The *p*‐value is derived from the Log Rank test; the *q*‐value is derived from the Benjamini–Hochberg FDR correction procedure. Each group's median months in survival are presented with a 95% CI

The primary aim of this study was to determine if the TFIIIB subunit BDP1 is specifically altered in ovarian cancer and if the observed alterations correlate with clinical outcomes. Using the cBioPortal platform,^38^, ^39^ we queried the TCGA Ovarian Serous Cystadenocarcinoma dataset (TCGA, Firehose Legacy),^40^ *n* = 594 patients, for BDP1 alterations. The dataset analysis identified 23 cases of BDP1 homodeletions (3.95% alteration frequency) and one case of BDP1 amplification (0.167% alteration frequency), Figure 1A. Upon further analysis, deep deletions of BDP1 correlate with decreased BDP1 expression in ovarian cancer, *n* = 538 samples, Figure 1B. As a result of this, we analyzed alterations and survival in serous ovarian cancer and found that BDP1 homodeletions and decreased expression negatively impacted disease‐free progression in patients (*p* = 0.0271, *q* = 0.0542), Figure 1C. Based on this data, we further investigated the individual patients with BDP1 alterations and disease‐free events in Figure 1C (data provided in Table S1). We examined age, disease‐free months, race, and stage and found the average age of the serous ovarian cancer patients with disease‐free events is 58.5 years old and the median disease‐free survival is 13.30 months (95% CI), Figure 1C. Most patients with shortened disease‐free progression with BDP1 alterations had stage IIIC and stage IV serous ovarian cancer diagnosis in agreement with prior observations that most serous carcinomas are diagnosed at stage III (51%) or IV (29%).^51^ Furthermore, the patients with BDP1 alterations and shortened disease‐free progression were 93.8% white. It is important to note that within the TCGA Ovarian Serous Cystadenocarcinoma dataset (TCGA, Firehose Legacy),^40^ the racial composition of the dataset is classified as 83.0% White, 5.7% Black or African‐American, 3.3% Asian, 0.5% American Indian or Alaskan Native and 0.2% Native Hawaiian or Other Pacific Islander. Race data were not available for 7.3% of the patients and representation within the TCGA Ovarian Serous Cystadenocarcinoma dataset is in accordance with published incidence and mortality rates by race and ethnicity.^51^ The significant discrepancy in representation across various ethnic groups further supports the need to broaden representation within these datasets to further our understanding of this disease. Lastly, we did not find a statistically significant change in overall survival in patients with BDP1 mutations (data not shown).

Together, these data suggest BDP1 is not prognostic in serous ovarian cancer; however, the data presented in Figure 1 suggests that BDP1 alterations in serous ovarian cancer warrant further investigation. We sought to determine if the observed alterations in BDP1 in serous ovarian cancer are unique to BDP1 (Figure 1) or are a common feature in all TFIIIB subunits including, BRF1, BRF2, and TBP which have been previously shown to be deregulated in cancer.

### 3.2 Analysis of serous ovarian cancer demonstrates a significant decrease in BDP1 mRNA expression

**FIGURE 2:**
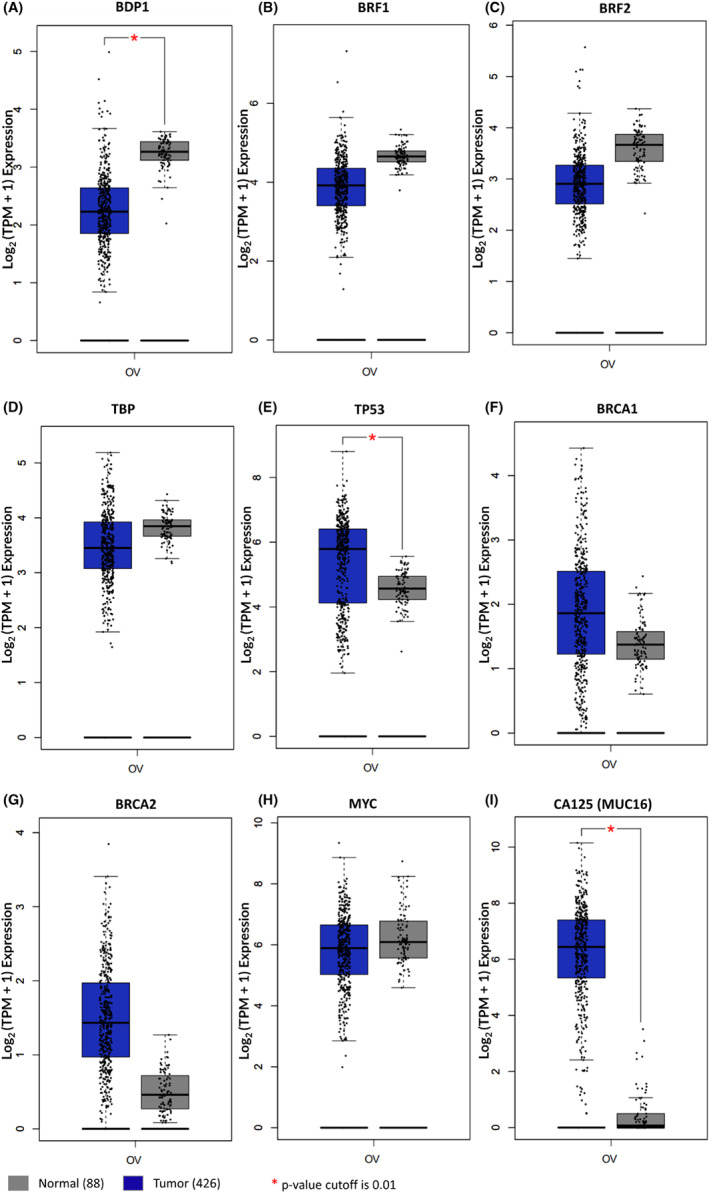
BDP1 expression is significantly decreased in ovarian cancer. We queried the Gene Expression Profiling Interactive Analysis (GEPIA)^43^, ^44^ to evaluate mRNA expression in ovarian cancer samples compared to control samples from the Cancer Genome Atlas (TCGA)^40^ and Genotype‐Tissue Expression (GTEx)^45^ project. mRNA expression of BDP1 (A), BRF1 (B), BRF2 (C), TBP (D), TP53 (E), BRCA1 (F), BRCA2 (G), MYC (H), and CA125 (I) are presented. The number of tumor samples analyzed was 426, and 88 normal samples for all analyses. The log_2_FC cutoff is 1.0, and the *p*‐value cutoff is 0.01 with statistical significance denoted. The matched normal analysis was performed using TCGA tumors versus TCGA normal and GTex normal. The log_2_ (TPM + 1) transformed expression data was used for plotting. Normal samples are denoted as gray boxes; tumors are depicted as blue boxes

The data presented in Figure 1 prompted further analysis into the mRNA expression of the TFIIIB subunits, BRF1, BRF2, and TBP in serous ovarian cancer, Figures 2A‐D. Using the Gene Expression Profiling Interactive Analysis (GEPIA) platform,^43^, ^44^ ovarian cancer samples from the Cancer Genome Atlas (TCGA) public dataset,^40^ *n* = 426, were compared to control samples from the Cancer Genome Atlas (TCGA)^40^ and Genotype‐Tissue Expression (GTEx) project,^45^ *n* = 88. Figure 2A demonstrates that BDP1 mRNA expression was observed to be significantly decreased in ovarian cancer, *p* = 0.01; however, the TFIIIB subunits BRF1, BRF2, and TBP mRNA expression was not significantly altered in ovarian cancer, Figure 2B‐D. It is well documented that TP53,^10^, ^11^ MYC,^11^, ^20^, ^52^ and BRCA1^9^ regulate RNA pol III transcription through TFIIIB and have been identified as regulators of ovarian cancer. Based on the previous results, we sought to determine if these regulators exhibited altered mRNA expression in the datasets analyzed for TFIIIB subunit mRNA expression. TP53, the most frequently mutated gene in cancer has been demonstrated to have a driver role in high‐grade serous ovarian cancer.^53^ Analysis using GEPIA identifies TP53 as significantly overexpressed in serous ovarian cancer, *p* = 0.01, Figure 2E and the individual patient data used for analysis of disease‐free progression in patients with BDP1 mutations (Figure 1C) shows that 62% of patients recorded with disease‐free events had a mutation in TP53 (data not shown). BRCA1 and BRCA2 alterations are frequently observed in familial and sporadic serous ovarian cancer^7^, ^54^ with approximately 15% of serous ovarian cancer patients exhibiting BRCA germline mutations.^55^ Specifically, BRCA1 has been shown to negatively regulate RNA pol III transcription via TFIIIB.^8^ Analysis of the GEPIA platform demonstrates that both BRCA1 (Figure 2F) and BRCA2 (Figure 2G) mRNA are overexpressed in serous ovarian cancer, but this overexpression is not statistically significant. Another well‐known driver of ovarian cancer, MYC, is amplified in approximately 50% of high‐grade serous ovarian cancer^56^; however, analysis of the GEPIA platform, using the TCGA and normal datasets did not identify MYC as significantly overexpressed, Figure 2H. Lastly, according to previously published reports,^2^, ^3^ serum CA‐125 levels are significantly elevated in the TCGA Ovarian Serous Cystadenocarcinoma dataset. The data presented in Figure 2I demonstrates a significant increase in CA125 expression in tumors, in agreement with previous findings.

Overall, although the TFIIIB subunits BRF2^16^, ^24^, ^25^, ^26^, ^28^, ^29^, ^30^, ^32^, ^34^, ^35^ and BRF1^20^, ^21^, ^23^, ^24^, ^57^ have been demonstrated to be deregulated in a variety of human cancers, the data presented in Figure 2 suggest that BDP1 is the only TFIIIB subunit specifically altered in serous ovarian cancer. However, the data in Figure 2 does not indicate whether BDP1 is specifically altered by stage in serous ovarian cancer.

### 3.3 Correlation between BDP1 mRNA expression and serous ovarian cancer by stage

**FIGURE 3:**
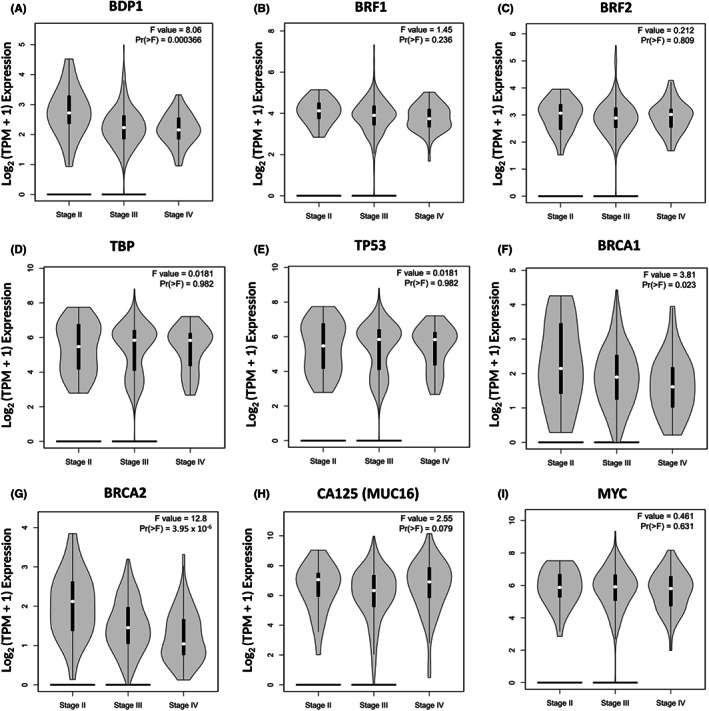
BDP1 mRNA expression correlates with serous ovarian cancer by stage. We queried the Gene Expression Profiling Interactive Analysis (GEPIA)^43^, ^44^ to evaluate if mRNA expression correlates with stages II, III and IV in serous ovarian cancer samples, compared to control, from the Cancer Genome Atlas (TCGA)^40^ and Genotype‐Tissue Expression (GTEx)^45^ project: BDP1 (A), BRF1 (B), BRF2 (C), TBP (D), TP53 (E), BRCA1 (F), BRCA2 (G), CA125 (H), and MYC (I). The matched normal analysis was performed using TCGA tumors versus TCGA normal and GTEx normal. The log_2_ (TPM + 1) transformed expression data were used for plotting; a one‐way ANOVA analysis was performed. F and Pr(>F) values are denoted for each gene and statistical significance is classified as Pr(>F) = 0.05. The pathological stage classification (II, III and IV) is based on the TCGA clinical annotation^44^, ^46^, ^47^

In Figure 2, we demonstrate that the TFIIIB subunit BDP1 is specifically decreased (*p* = 0.01) in serous ovarian cancer and we wanted to determine if this alteration is stage‐speciifc in serous ovarian cancer. We queried the GEPIA platform using the TCGA serous Ovarian Serous Cystademocarcinoma and GTEx gene expression datasets to analyze BDP1 expression across stages in serous ovarian cancer.^43^, ^44^ Figure 3 presents BDP1 expression at stages II, III and IV using violin plots comparing TCGA tumor data to TCGA and GTEx normal data. The GEPIA platform utilizes pathological stage classification based on the TCGA clinical annotation.

Figure 3 shows that BDP1 expression significantly decreased in serous ovarian cancer as ovarian stage increased (F = 8.06; Pr(>F) = 0.000366), Figure 3A. In line with the results presented in Figures 2B‐D, we observed no significant expression changes in the TFIIIB subunits BRF1 (Figure 3B), BRF2 (Figure 3C), or TBP (Figure 3D). We further analyzed additional regulators of RNA pol III transcription as well to determine if there was a significant correlation with expression and stage. TP53, although frequently mutated in serous ovarian cancer,^53^ did not have a statistically significant change in expression by ovarian cancer stage, Figure 3E. BRCA1 and BRCA2 mutations, previouslyidentified in both sporadic and hereditary serous ovarian cancer,^6^, ^54^ had significant decreases in the TCGA Ovarian Serous Cystadenocarcinoma dataset, BRCA1 (F = 3.81; Pr(>F) = 0.023) and BRCA2 (F = 12.8; Pr(>F) = 3.95 x 10^−6^), Figure 3F‐G. CA125 (MUC16*)* expression increased (F = 2.55; Pr(>F) = 0.079) with serous ovarian cancer stage as previously reported,^3^ but not significantly, Figure 3H. Lastly, MYC did not exhibit significant increases in the TCGA Ovarian Serous Cystadenocarcinoma dataset, Figure 3I. Together, these data suggest that only BDP1 of the TFIIIB complex has expression correlating with stages II, III and IV in serous ovarian cancer (Figure 3A) which interestingly, is similar to BRCA1 (Figure 3F) and BRCA2 (Figure 3G), established drivers of serous ovarian cancer.^6^, ^53^

### 3.4 Overall and progression‐free survival in serous ovarian cancer is affected by BDP1 expression

**FIGURE 4:**
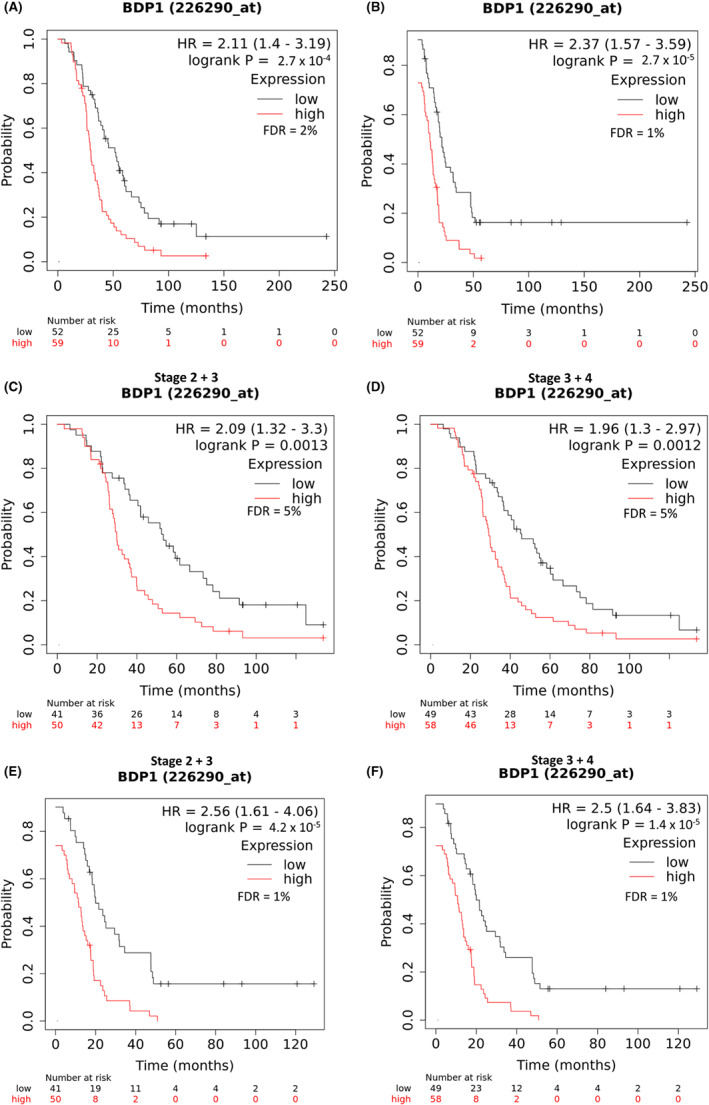
Survival of patients in serous ovarian carcinoma with BDP1 alterations and TP53 mutations. The Kaplan–Meier Plotter^48^ web portal was used to analyze BDP1 expression and survival in serous ovarian cancer in samples containing TP53 mutations. (A) High BDP1 expression is unfavorable for overall survival, *n* = 111; *p* = 2.7 *×* 10^−4^; HR = 2.11. The median overall survival for high BDP1 expression was 29.9 months and 51.6 months for low BDP1 expression. (B) High BDP1 expression is unfavorable for progression‐free survival, *n* = 111; *p* = 2.7 *×* 10^−5^; HR = 2.37. The median progression‐free survival for high BDP1 expression was 10.8 months and 21.6 months for low BDP1 expression. (C) High BDP1 expression is unfavorable for overall survival in stages 2 and 3, *n* = 91; *p* = 0.0013; HR = 2.09. The median survival for high BDP1 expression was 29.9 months and 53.3 months for low BDP1 expression. (D) High BDP1 expression is unfavorable for overall survival in stages 3 and 4 serous ovarian cancer, *n* = 107; *p* = 0.0012; HR = 1.96. The median survival for high BDP1 expression was 29.23 months and 45.77 months for low BDP1expression. (E) High BDP1 expression is unfavorable for progression‐free survival in stages 2 and 3, *n* = 91; *p* = 4.2 *×* 10^−5^; HR = 2.56. The median progression‐free survival for high BDP1 expression was 11.3 months and 19.98 months for low BDP1 expression. (F) High BDP1 expression is unfavorable for progression‐free survival in stages 3 and 4, *n* = 107; *p* = 1.4 *×* 10^−5^; HR = 2.5. The median progression‐free survival for high BDP1 expression was 10.68 months and 19.88 months for low BDP1 expression. False discovery rates (FDR) are noted

The significant decrease in BDP1 (Figure 2A) and its stage‐specific decrease (Figure 3A) prompted a query of BDP1 expression in overall and progression‐free survival. Disease‐free progression is defined as the time a patient survives after primary treatment without cancer symptoms and is useful in determining the effectiveness of new therapies, especially in identifying and characterizing biomarkers. Overall, survival is defined as the time from diagnosis to death and may include multiple treatments. Using the Kaplan–Meier Plotter^48^ web portal, we analyzed both BDP1 expression and survival in serous ovarian cancer. As shown in Figure 1C, BDP1 alterations negatively impacted disease‐free progression in patients with ovarian cancer (*p* = 0.0271, *q* = 0.0542). Additional analysis of the patients and progression‐free survival events identified 62.5% of patients having a mutation(s) in TP53 (data not shown). Thus, we chose to analyze BDP1 expression in the context of TP53 mutations. Interestingly, analysis of BDP1 in samples that also contain TP53 mutations, high BDP1 expression appears to be unfavorable for overall survival, *n* = 111; *p* = 2.7 × 10^−4^; Hazard Ratio (HR) = 2.11, Figure 4A. The median overall survival for high BDP1 expression was 29.9 months and 51.6 months for low BDP1 expression, with a 2% FDR. In addition, high BDP1 expression is unfavorable for progression‐free survival, *n* = 111; *p* = 2.7 × 10^−5^; HR = 2.37, Figure 4B. The median progression‐free survival for high BDP1 expression was 10.8 months and 21.6 months for low BDP1 expression, and a FDR of 1% was calculated. In Figure 3A, we show that the TFIIIB subunit BDP1 exhibited significantly decreased expression in serous ovarian cancer as stage increased. In contrast with this previously presented data, Figure 4 demonstrates that consideration of the overall mutational profile of the cancer must be considered as it could potentially contribute to overexpression of BDP1 through a regulatory network, demonstrating a possible dual role for BDP1 in serous ovarian cancer that is dependent on the overall mutational profile.

**FIGURE 5:**
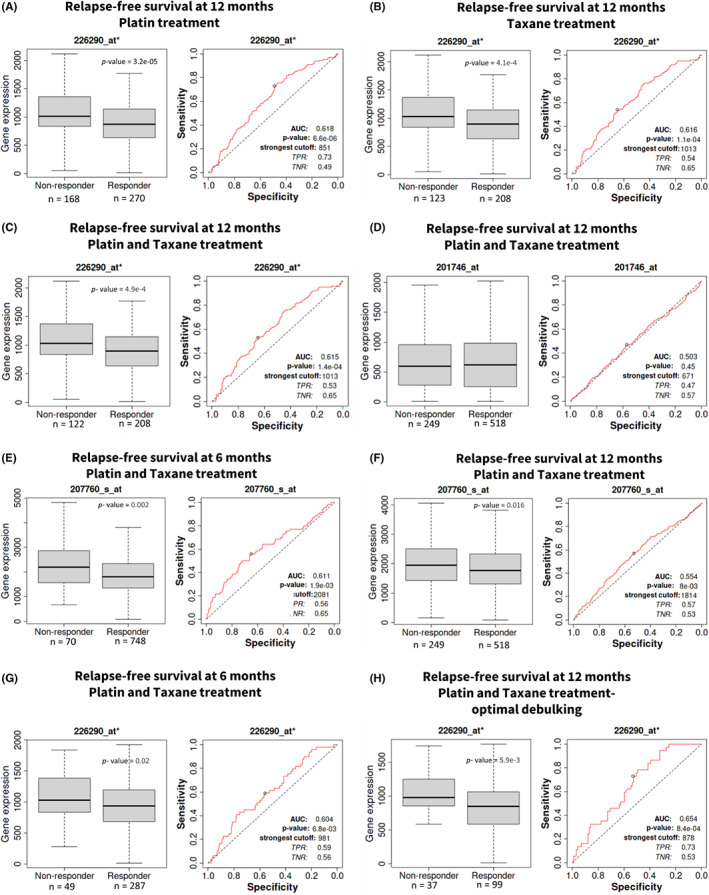
Analysis of BDP1 as a candidate predictive biomarker in serous ovarian cancer. ROC analysis of BDP1 (probe 226290_at*) expression and specificity twelve‐months relapse‐free in response to (A) platin, (B) taxane, and (C) platin and taxane treatment. As a comparison, TP53 (probe 201746_at) (D) expression in response to platin and taxane treatment in serous ovarian cancer is presented. NCOR2 (probe 207760_s_at) expression demonstrates a significant correlation with response at six (E) and twelve (F) months of combination platin and taxane treatment. Like NCOR2 (E), BDP1 (probe 226290_at*) expression demonstrates a significant correlation with response at six months of combination platin and taxane treatment (G). Restricting serous ovarian cancer patient samples to optimimized debulking significantly increased the AUC score for BDP1 (probe 226290_at*) (H). Responders and nonresponders were compared using the Mann–Whitney test, and significant *p*‐values are denoted. The area under the curve (AUC) and associated *p*‐values are depicted. The cutoff for p values was set at *p* < 0.05, and only results with a 5% false discovery rate (FDR) were considered significant

We next examined survival, relative to BDP1 expression and TP53 mutations, by serous ovarian cancer stages. In samples containing TP53 mutations, high BDP1 expression is unfavorable for overall survival in stages II and III, *n* = 91; *p* = 0.0013; HR = 2.09, 5% FDR (Figure 4C) and the median survival for high BDP1 expression was 29.9 months. In these samples with TP53 mutations and low BDP1 expression, median survival was 53.3 months (Figure 5C). In stages III and IV, Figure 4D, high BDP1 expression is unfavorable for overall survival, *n* = 107; *p* = 0.0012; HR = 1.96, 5% FDR and the median survival was 29.23 months. In these samples with low BDP1 expression, median survival was 45.77, Figure 4D The analysis for progression‐free survival demonstrates that high BDP1 expression is unfavorable for in stages II and III, *n* = 91; *p* = 4.2 × 10^−5^; HR = 2.56; 1% FDR, Figure 4E, and the median progression‐free survival for high BDP1 expression was 11.3 months. In these samples with low BDP1 expression, the median progression‐free surival was 19.98 months, Figure 4E. In stages III and IV, high BDP1 expression is unfavorable for progression‐free survival, *n* = 107; *p* = 1.4 × 10^−5^; HR = 2.5; 1% FDR, Figure 4F, and the median progression‐free survival for high BDP1 expression was 10.68 months. In these samples with low BDP1 expression, the median progression‐free survival was 19.88 months, Figure 4F. Together, these data suggest that further investigations of BDP1 expression in serous ovarian cancer are warranted, specifically in the context of additional muations such as TP53 to determine how dual‐expression of BDP1 potentially contributes to serous ovarian cancer.

### 3.5 BDP1 as a predictive biomarker in serous ovarian cancer

Figure 4 provides evidence of high BDP1 expression negatively impacting overall (Figure 4A) and progression‐free (Figure 4B) survival in serous ovarian cancer in samples containing TP53 mutations. Further, our analysis shows that in the context of TP53 mutations, high BDP1 expression is unfavorable for progression‐free survival in stages II and III (Figure 4C) and stages III and IV (Figure 4D). This high expression of BDP1 correlating with poor survival is in contrast with the observed decrease of BDP1 expression in serous ovarian cancer presented in Figure 2 and the negative BDP1 expression correlating with increased stage in serous ovarian cancer as demonstrated in Figure 3. We believe this differential expression of BDP1 and its correlation with stage and survival warrants further investigation regarding the possibility of BDP1 as a predictive biomarker. Thus, we investigated the possibility of BDP1 as a predictive biomarker for chemotherapy treatment in serous ovarian cancer using the ROC plotter platform.^49^ The ROC Plotter platform can identify potential predictive biomarkers which could predict the response to the most commonly used combination treatment, platin, and taxane, in serous ovarian tumors.^49^ We performed a ROC analysis and Mann–Whitney tests for BDP1 on serous ovarian cancer samples treated with first‐line chemotherapeutic agents^58^ (Figure 5). It is well established that half of ovarian cancer recurrences occur at more than twelve months from the start of therapy, and one‐quarter of all ovarian cancer recurrences occur within six months of first‐line treatment.^59^ We analyzed BDP1 expression in response to common serous ovarian cancer chemotherapies and at twelve‐month relapse‐free survival in patients treated with platin (Figure 5A), BDP1 expression significantly decreased (*p* = 3.2 × 10^−05^). The ROC analysis (*p* = 6.6 × 10^−06^, AUC = 0.618) suggests BDP1 may be a predictor of a serous ovarian cancer patient's response to platin‐based chemotherapy. Similarily, BDP1 expression significantly decreased (*p* = 4.1 × 10^−4^) in ovarian cancer patient's treated with taxane, and ROC analysis (*p* = 1.1 × 10^−04^, AUC = 0.616). Figure 5C demonstrates that a combination of both platin and taxane also result in a significant decrease of BDP1 expression (*p* = 1.4 × 10^−04^, AUC = 0.615). In line with consideration of BDP1 expression in the context of TP53 mutations, we also analyzed TP53 expression for relapse‐free survival at 12 months in response to both platin and taxane in serous ovarian cancer, Figure 5D, to determine its status as a predictive biomarker. This analysis suggests there is no statistical significance in TP53 gene expression (*p* = 0.45, AUC = 0.503), and that TP53 alone is not predictive. Additionally, Fekete et al.^49^ identified NCOR2 as a predictive biomarker of serous ovarian cancer for both platin and taxane treatment during development of the ROC Plotter platform. The identification of NCOR2 as one of the top eight genes responding to combination therapy in ovarian cancer is significant because like BDP1, NCOR2 contains a SANT domain (SWI3, ADA2, N‐Cor, and yeast TFIIIB BDP1 proteins) (reviewed in^17^). Specifically, NCOR2 was one of the most significant genes validated in their study^49^ and our analysis, Figure 5E‐F, demonstrates that NCOR2 at both 6‐ and 12‐month relapse‐free survival for platin and taxane treatment is significantly decreased, *p* = 0.002 and *p* = 0.016, with AUC = 0.611 and AUC = 0.554, respectively. When comparing NCOR2 predictiveness to BDP1 at 6‐months for platin and taxane, Figure 5G, BDP1 expression significantly decreases, *p* = 0.02, which is comparable to NCOR2 at 6‐months (Figure 5E). For 12‐months relapse‐free survival, BDP1 (Figure 5A) is a stronger predictor, AUC = 0.618, of combination treatment response in comparison to NCOR2 (Figure 5F), AUC = 0.554. Lastly, in an additional analysis for BDP1 expression which restricts the samples to those patients that received both platin and taxane treatment after optimal debulking surgery, the AUC score for BDP1 was significantly increased, AUC = 0.654 (Figure 5H). Taken together, this suggests that BDP1 is behaving similarly to an already established biomarker of serous ovarian cancer, NCOR2, in clinical samples treated with chemotherapies platin and taxane. These data support that BDP1 may be a predictor of a serous ovarian cancer patient's response to taxane‐based chemotherapy.

## 4 DISCUSSION

Recently, BDP1 alterations in human cancers have been identified,^34^, ^35^, ^36^ including BDP1 somatic frameshift mutations in colorectal cancer, *n =* 98 and two BDP1 variants associated with poor clinical outcomes in neuroblastoma.^36^ Most recently, we have shown BDP1 expression has been correlated with clinical outcomes in non‐Hodgkin lymphoma^35^ and breast cancer^34^ as well. These recent BDP1 clinical cancer studies prompted our investigation of BDP1 alterations and expression in ovarian cancer. These analyses demonstrate that BDP1 alterations in ovarian cancer are mostly deep deletions (3.95%), correlate with decreased expression in serous ovarian carcinoma (Figure 1A‐B) and that BDP1 alterations negatively impacted disease‐free progression in patients with ovarian cancer (*p* = 0.0271, *q* = 0.0542) (Figure 1C).

It is well documented that TP53,^10^, ^11^ MYC,^11^, ^20^, ^52^ and BRCA1^9^ regulate RNA pol III transcription through TFIIIB. Both BRCA1 and BRCA2 mutations have been previously identified in sporadic and hereditary serous ovarian cancer.^6^, ^54^ Using the cBioPortal and the same TCGA dataset, we note that BRCA1 (4%) and BRCA2 (5%) are altered in serous ovarian cancer, and the alterations include amplifications and homodeletions. In the case of BRCA1, these alterations do not significantly alter overall survival or disease‐free progression, but BRCA2 alterations correlate with a decrease in overall survival and not disease‐free progression (data not shown). Both MYC (42%) and TP53 (49%) are altered in ovarian cancer patients in the same dataset we profiled BDP1, but these alterations do not significantly alter overall survival or disease‐free progression (data not shown). Taken together, this potentially suggest a key role for BDP1 in serous ovarian cancer.

BDP1 mRNA expression was significantly decreased in ovarian cancer, *p* = 0.01 (Figure 2A) but the expression of other TFIIIB subunits, BRF1 (Figure 2B), BRF2 (Figure 2C), and TBP (Figure 2D), was not significantly changed. TP53 expression was significantly increased in ovarian cancer (Figure 2E). However, the expression of the tumor suppressors BRCA1 (Figure 2F) and BRCA2 (Figure 2G) were not significantly altered in ovarian cancer. Only the TFIIIB subunit BDP1 exhibited significantly decreased expression in serous ovarian cancer as the stage increased (F = 8.06; Pr(>F) = 3.66 *×* 10^−4^) (Figure 3A). Interestingly, BDP1 expression decreased as serous ovarian cancer stage similar to BRCA1 (F = 3.81; Pr(>F) = 2.3 *×* 10^−2^) and BRCA2 (F = 12.8; Pr(>F) = 3.95 *×* 10^−6^) (Figure 3F,G). Previously, we demonstrated that BRCA1 negatively regulates RNA pol III transcription via TFIIIB^9^ and RNA pol III transcription has been linked to double‐stranded DNA‐damage repair.^60^ Together, these data suggest potential cross‐talk between BRCA1 and BDP1 activity in late‐stage serous ovarian cancer as a component of the oncogenic network driving proliferation.

TP53 mutations are prevalent in many human cancers, including ovarian cancer.^53^ Interestingly, our analysis suggest that in serous ovarian cancer patients with TP53 mutations, high BDP1 expression is unfavorable for overall survival, *n* = 111; *p* = 2.7 *×* 10^−4^; Hazard Ration (HR) = 2.11, with a 2% FDR (Figure 4A), is unfavorable for overall survival in stages II and III, *n* = 91; *p* = 0.0013; HR = 2.09, *5%* FDR (Figure 4C), and III and IV *n* = 107; *p* = 0.0012; HR = 1.96, *5%* FDR *(*Figure 4D *)*. Additionally, high BDP1 expression is disadvantageous for progression free survival, *n* = 111; *p* = 2.7 *×* 10^−5^; HR = 2.37, and a 1% FDR (Figure 4B), unfavorable for progression free survival in stages II and III, *n* = 91; *p* = 4.2 *×* 10^−5^; HR = 2.56; 1% FDR (Figure 4E) and in stages III and IV *n* = 107; *p* = 1.4 *×* 10^−5^; HR = 2.5; 1% FDR (Figure 4F).

These specific data, in contrast with the significant under expression of BDP1 seen in Figures 1 and 2, suggest a dual role for BDP1 in the biosynthetic capacity of a cell. BDP1 could potentially display both oncogenic and tumor‐suppressing function that is dependent on its mutational status and can vary based on the unique mutational profile of the cancer. Homodeletions of BDP1 could result in elimination of its tumor suppressing capability whereas overexpression can contribute to its oncogenic property and could be driven by major mutations such as TP53 which has been previously shown to regulate TFIIIB. Recently, Shen et al^61^ demonstrated that genes classified with this dual function in 12 major cancer types, including ovarian serous carcinoma, termed “double‐agent” genes, are mainly classified as transcription factors that can both positively and negatively affect transcription. Specifically, they identified ovarian cancer as having over‐representation of these types of dually expressed genes.^61^ Given the role of BDP1 as a component of both of forms of TFIIIB and its role in interaction with TFIIIC, it is possible either its under‐ or over‐expression could contribute to aberrant proliferation and is dependent on the type of mutation incurred.

A recent model of TFIIIC‐directed assembly of TFIIIB suggests that TFIIIC interacts with BRF1, drives the recruitment of TBP and lastly, BDP1, via interactions with the TFIIIC‐Tfc4 and ‐Tfc8 subunits, respectively.^62^ Furthermore, the model suggests that BDP1 recruitment results in displacement of the τB module of TFIIIC, driving TFIIIC dissociation from the gene. As BRF1 is the primary TFIIIB subunit necessary for TFIIIC contact and in turn, TBP recruitment, in a cellular environment that has acquired mutations in known modulators of RNA pol III specific transcription, it is plausible that the recruitment of BRF1 and TBP alone by TFIIIC may be sufficient to drive transcription without the need to recruit the last TFIIIB component, BDP1. This could potentially account for the ability of cancers with BDP1 homodeletions to continue with RNA pol III transcription in its absence as these regulators would have direct interaction with an already formed BRF1‐TBP complex. Additionally, it has been demonstrated that BDP1 phosphorylation by CK2 inactivates pol III transcription during mitosis by resulting in dissociation from the chromatin but, BRF1 and TBP remain associated.^63^, ^64^ Thus, in a cellular environment with BDP1 homodeletions, it is possible that the BRF1‐TBP complex could still form and remain associated with the DNA and additional acquired mutations could directly regulate BRF1‐ TBP. It is plausible this is sufficient to continue driving RNA pol III transcription, contributing to uncontrolled proliferation. Conversely, in a cellular environment with high BDP1 expression, over activity of TFIIIB by increased BDP1 levels would drive increased RNA pol III transcription as well. Previously, Winter et al demonstrated that RNA pol III specific products, tRNA, 5 s rRNA and 7SL RNAs are overexpressed in ovarian tumors compared to normal ovarian tissue.^65^ As BDP1 overexpression would contribute to these increased levels, the overexpression data presented here is in line with this previous observation.^65^

In line with a dual role for BDP1 dependent on the mutational profile of the cancer, we recognize that ovarian cancer cells depend on MYC for maintaining their oncogenic growth and is amplified in 30–60% of all ovarian cancers.^66^ MYC has been shown to interact with SP1 to decrease cyclin‐dependent kinase inhibitor (p21) gene expression,^67^ therefore, we speculated that BDP1 gene expression may be specifically decreased through MYC/SP1 interactions. We queried the Eukaryotic Promoter Database (https://epd.epfl.ch//index.php) for putative SP1 binding sites in the BDP1promoter^68^ and identified putative SP1 binding sites within the BDP1 promoter at: −897, −738, −521, −403, −360, −17, −4, 52, 63, and 80, relative to the transcriptional start site (TSS) (+1), *p*‐value of 0.001, (https://epd.epfl.ch//index.php, accessed March – April 2022). This decreased expression of BDP1 by a larger oncogenic regulatory network could result in its inability to carry out its tumor suppressing capabilities.

Half of ovarian cancer recurrences occur at more than twelve months from first diagnosis.^59^ Thus, we investigated BDP1 expression in response to common serous ovarian cancer chemotherapies. Fekete et al. identified the top eight predictive biomarker candidates responding to the most common serous ovarian cancer treatment combination of platin and taxane.^49^ The list of top eight genes identified include the nuclear receptor corepressor 2 (NCOR2) (*p* = 1.90 *×* 10^−03^, AUC = 0.611), the translocation of the transcription factor E3 (TFE3) (*p* = 7.90 *×* 10^−05^, AUC = 0.631), and the pyridoxal kinase (PDXK) (*p* = 1.40 *×* 10^−04^, AUC = 0.634).^49^ The identification of NCOR2 as one of the top eight genes responding to combination therapy in ovarian cancer is significant because like BDP1, NCOR2 contains a SANT domain. Thus, we sought to determine if BDP1 is a potential predictive biomarker in ovarian cancer, In Figure 5, we analyzed BDP1 expression at twelve‐month relapse‐free survival in patients treated with platin (Figure 5A), taxane (Figure 5B), or combination therapy (Figure 5C). Figure 5 demonstrates BDP1 expression is significantly decreased in all chemotherapeutics tested and importantly, all BDP1‐related ROC analysis presented in response to chemotherapy were significant with AUC values greater than 0.6, suggesting BDP1 may be a biomarker with clinical potential. Furthermore, TP53 is frequently mutated in ovarian cancer and ROC analysis (Figure 5D) did not suggest a role for TP53 as a predictive biomarker in ovarian cancer. The additional analysis for NCOR2 expression in response to combination therapy at both six (Figure 5E)^49^ and twelve (Figure 5F) months shows a significant decrease in expression. However, at twelve months relapse‐free survival in response to combination therapy, ROC analyses identified more significant AUC outcomes for BDP1 (Figure 5C) than NCOR2 (Figure 5F). Using the six‐month relapse‐free endpoint, both NCOR2 (Figure 5E) and BDP1 (Figure 5G) had similar outcomes in response to combination therapy. Interestingly, applying a debulking optimization in the context of combination treatment, using a twelve‐month relapse‐free endpoint, the ROC analyses outcomes are more striking, AUC = 0.65 (Figure 5H). Figure 5 suggests additional studies investigating BDP1 as a predictive ovarian cancer biomarker in the clinic are warranted.

Cisplatin has been demonstrated to disrupt ERK, MAPK, TP53, and JNK signaling,^69^ and these pathways have been demonstrated to regulate TFIIIB‐mediated transcription.^10^, ^20^, ^63^, ^70^, ^71^, ^72^ At twelve‐month relapse‐free survival, in patients treated with platin, BDP1 expression significantly decreased (*p* = 0.0014), and the ROC analysis (*p* = 7.6 *×* 10^−05^, AUC = 0.663) (Figure 5A) suggest BDP1 could be of clinical relevance as a predictive biomarker in serous ovarian cancer.

Taxanes regulate microtubule assembly, induce TP53, and inhibit various cyclin‐dependent kinases (CDKs).^73^ Previously, it has been demonstrated that microtubule association is required for gene external (tRNA) RNA pol III transcription^74^ and TFIIIB‐mediated transcription is modulated by TP53 and CDKs.^14^, ^15^, ^63^ Interestingly, Ying Yang 1 (YY1) modulates taxane response in serous ovarian cancer^75^ and a query of the Eukaryotic Promoter Database^68^ for putative *YY1* binding sites in the BDP1 promoter show there are two putative *YY1* binding sites within the BDP1 promoter at −94 and − 68, relative to the transcriptional start site (TSS) (+1), with a *p*‐value cutoff of 0.001 (https://epd.epfl.ch//index.php, accessed April 2022). At twelve‐month relapse‐free survival, in patients treated with taxane, BDP1 expression significantly decreased (*p* = 0.0059), and the ROC analysis (*p* = 8.4 *×* 10^−04^, AUC = 0.654) (Figure 5B) suggest BDP1 may be of predictive value in patients with serous ovarian cancer.

To gain additional insight regarding the network BDP1 alterations affect in the context of ovarian cancer, we performed a gene ontology analysis of genes co‐expressed with BDP1. Using the cBioPortal,^38^ we performed an analysis for genes co‐expressed with BDP1 in the TGCA ovarian cancer dataset, using Spearman's correlation coefficient cutoff value of 0.5 (Table 2).

**TABLE 2:** Genes significantly co‐expressed with BDP1 in serous ovarian cancer

Correlated Gene	Cytoband	Spearman's Correlation	*p‐Value*	*q‐Value*
MTX3	5q14.1	0.691918711	4.65E‐45	9.29E‐41
SREK1	5q12.3	0.669282677	3.06E‐41	3.05E‐37
RAD17	5q13.2	0.592933889	1.56E‐30	1.04E‐26
CHD1	5q15‐q21.1	0.591815348	2.13E‐30	1.07E‐26
TNPO1	5q13.2	0.588823657	4.90E‐30	1.96E‐26
ARHGEF28	5q13.2	0.585609045	1.19E‐29	3.95E‐26
AGGF1	5q13.3	0.573440511	3.09E‐28	8.82E‐25
GUSBP3	5q13.2	0.559088246	1.22E‐26	3.04E‐23
GOLGA2P5	12q23.1	0.556437824	2.35E‐26	5.22E‐23
FNBP4	11p11.2	0.551602427	7.71E‐26	1.54E‐22
ZSWIM6	5q12.1	0.549366383	1.33E‐25	2.41E‐22
UTP15	5q13.2	0.547246258	2.21E‐25	3.68E‐22
LUC7L3	17q21.33	0.540838808	1.01E‐24	1.56E‐21
ZFC3H1	12q21.1	0.539688955	1.33E‐24	1.89E‐21
MCCC2	5q13.2	0.537847946	2.04E‐24	2.72E‐21
NKTR	3p22.1	0.536800325	2.60E‐24	3.25E‐21
ERBIN	5q12.3	0.531072834	9.69E‐24	1.14E‐20
PPWD1	5q12.3	0.530814868	1.03E‐23	1.14E‐20
GTF2H2C	5q13.2	0.52574805	3.22E‐23	3.38E‐20
POLK	5q13.3	0.523485463	5.32E‐23	5.32E‐20
CELF1	11p11.2	0.518458044	1.61E‐22	1.53E‐19
TARDBP	1p36.22	0.515704825	2.93E‐22	2.66E‐19
MSH3	5q14.1	0.514910193	3.47E‐22	3.02E‐19
TRIM78P	11p15	0.513015896	5.22E‐22	4.35E‐19
SCAMP1	5q14.1	0.509702157	1.06E‐21	8.45E‐19
CPSF6	12q15	0.508255146	1.44E‐21	1.10E‐18
ZFYVE16	5q14.1	0.507648596	1.63E‐21	1.21E‐18
JMY	5q14.1	0.506312527	2.16E‐21	1.54E‐18
SLC30A5	5q13.1‐q13.2	0.504543889	3.13E‐21	2.15E‐18
PTCD2	5q13.2	0.502093635	5.20E‐21	3.46E‐18

**FIGURE 6:**
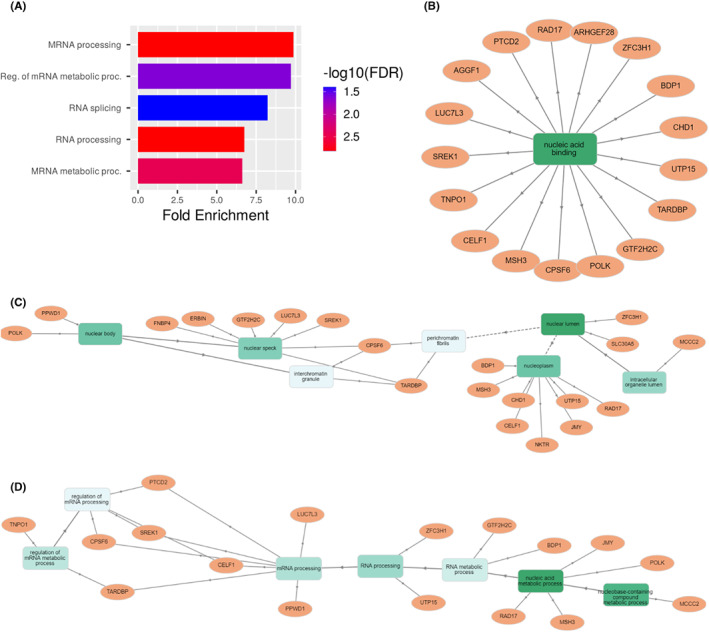
Gene ontology analysis of genes co‐expressed with BDP1in ovarian cancer. After querying the cBioPortal to identify genes co‐expressed with BDP1 (Spearman correlation threshold value of 0.5 and a statistically significant q‐value), we performed a gene ontology GO term annotation analysis. Using ShinyGO 0.76,^76^ accessed August 2022, we identified classes of biological processes BDP1 and significantly co‐expressed genes in serous ovarian cancer (A). The fold change enriched for each biological process is noted. FDR is reported as ‐log10(FDR). To drill down to gene‐term interactions, we used the open source GOnet web application^77^ to identify molecular functions (B), cellular localization (C), and (D) biological functions of genes co‐expressed with BDP1 in serous ovarian cancer. Genes are represented by circles; rectangles represent biological processes

Thirty genes were significantly co‐expressed with BDP1 in ovarian cancer based on the *q*‐value derived from the Benjamini–Hochberg FDR correction procedure and Spearman's coefficient cutoff value of 0.5. Next, we performed a gene ontology (GEO) enrichment analysis of genes identified in Table 2 to identify gene function and cell functions altered in serous ovarian cancer (Figure 6). In Figure 6A, we present the general cellular process altered, fold enriched and FDR. Overwhelmingly, the co‐expressed genes were involved in RNA metabolism and processes related to cell growth. These findings reflect prior observations implicating RNA pol III transcription, requiring TFIIIB, as a key mechanism dictating the biosynthetic capacity of a cell.^14^, ^15^ We used the open source GOnet web application^77^ to identify molecular functions (Figure 6B), cellular localization (Figure 6C), and (Figure 6D) biological functions for genes identified in Table 2 as significantly co‐expressed with BDP1 in serous ovarian cancer.

Together, our data suggest BDP1 expression is deregulated in serous ovarian cancer with clinical samples demonstrating BDP1 may be both over‐ and under‐expressed, suggesting dual function for BDP1. We recognize that conclusions from the analysis of large RNA‐seq datasets should always be interpreted cautiously. The scientific community needs to develop a standardized clinical data collection and reporting protocol for each sample analyzed.^78^ However, as presented, the current data generated from the analyzed clinical samples support a correlation with BDP1 expression and both overall and progression‐free survival. Further, BDP1 expression and survival are stage specific. Finally, BDP1 may have clinical applications to predict serous ovarian cancer response to platin and taxane, comparable to previously identified biomarkers of serous ovarian cancer. However, larger clinical studies are warranted to evaluate the clinical use of BDP1 as a predictive biomarker in serous ovarian cancer, especially by stage.

## AUTHOR CONTRIBUTIONS

**Stephanie Cabarcas‐Petroski:** Data curation (supporting); formal analysis (supporting); writing – original draft (supporting); writing – review and editing (lead). **Gabriella Olshefsky:** Data curation (supporting); formal analysis (supporting); validation (supporting); writing – review and editing (supporting). **Laura Schramm:** Conceptualization (lead); data curation (lead); formal analysis (lead); funding acquisition (lead); investigation (lead); methodology (lead); project administration (lead); writing – original draft (lead); writing – review and editing (equal).

## FUNDING INFORMATION

Not applicable.

## CONFLICT OF INTEREST

The authors declare no conflict of interest.

## INSTITUTIONAL REVIEW BOARD STATEMENT

Not applicable.

## INFORMED CONSENT STATEMENT

Not applicable.

## Supporting information


Table S1

## Data Availability

The present study used publicly available datasets archived in NCBI Gene Expression Omnibus and the cBioPortal. Hyperlinks to datasets are provided in the Methods section, Table 1.
