# Supplementary material for: BDP1 as a biomarker in serous ovarian cancer
Source: Cancer Med. 2022 Oct 28;12(5):6401–18. doi: 10.1002/cam4.5388 (PMC10028122; doi:10.1002/cam4.5388)
Supplement: Supplementary file 1 — Table S1 [file CAM4-12-6401-s001.docx]

**Supplemental Table 1.** Demographics and ovarian cancer stage of patients with BDP1 alterations analyzed in the BDP1 disease-free survival curve presented in Figure 1.

**
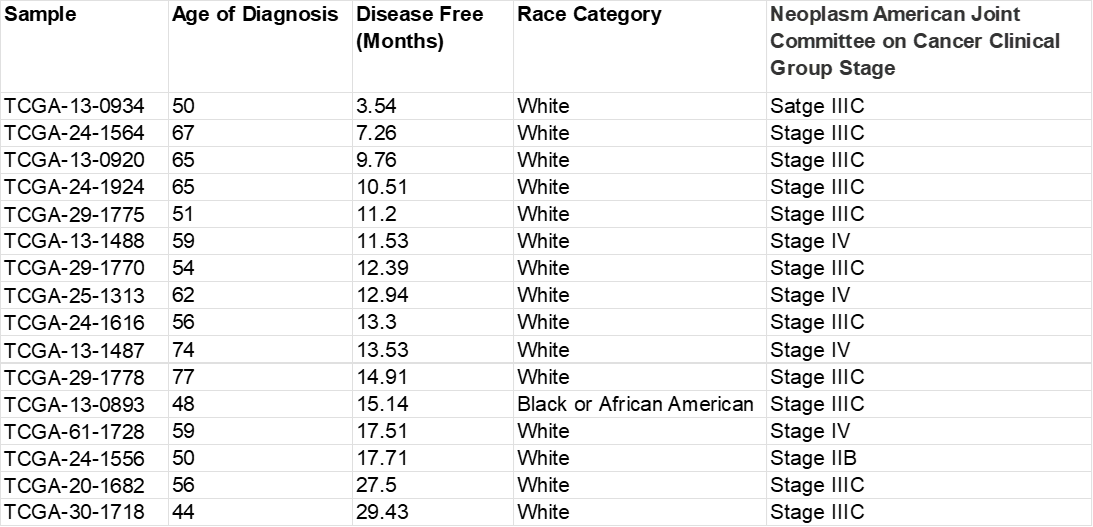
**
